# BUGSnet: an R package to facilitate the conduct and reporting of Bayesian network Meta-analyses

**DOI:** 10.1186/s12874-019-0829-2

**Published:** 2019-10-22

**Authors:** Audrey Béliveau, Devon J. Boyne, Justin Slater, Darren Brenner, Paul Arora

**Affiliations:** 1 Department of Statistics and Actuarial Science, University of Waterloo, 200 University Avenue West, Waterloo, Ontario N2L 3G1 Canada; 2 Division of Analytics, Lighthouse Outcomes, 1 University Avenue (3rd Floor), Toronto, Ontario M5J 2P1 Canada; 3 Department of Community Health Sciences, University of Calgary, 2500 University Drive NW, Calgary, Alberta T2N 1N4 Canada; 4 Department of Oncology, University of Calgary, 2500 University Drive NW, Calgary, Alberta T2N 1N4 Canada; 5 Dalla Lana School of Public Health, University of Toronto, Health Sciences Building, 155 College Street (6th Floor), Toronto, Ontario M5T 3M7 Canada

**Keywords:** Network meta-analysis, Indirect treatment comparison, Systematic review, Bayesian inference, Knowledge synthesis, Health technology assessment, Clinical efficacy, R package, Reporting guidelines

## Abstract

**Background:**

Several reviews have noted shortcomings regarding the quality and reporting of network meta-analyses (NMAs). We suspect that this issue may be partially attributable to limitations in current NMA software which do not readily produce all of the output needed to satisfy current guidelines.

**Results:**

To better facilitate the conduct and reporting of NMAs, we have created an R package called “BUGSnet” (**B**ayesian inference **U**sing **G**ibbs **S**ampling to conduct a **Net**work meta-analysis). This R package relies upon Just Another Gibbs Sampler (JAGS) to conduct Bayesian NMA using a generalized linear model. BUGSnet contains a suite of functions that can be used to describe the evidence network, estimate a model and assess the model fit and convergence, assess the presence of heterogeneity and inconsistency, and output the results in a variety of formats including league tables and surface under the cumulative rank curve (SUCRA) plots. We provide a demonstration of the functions contained within BUGSnet by recreating a Bayesian NMA found in the second technical support document composed by the National Institute for Health and Care Excellence Decision Support Unit (NICE-DSU). We have also mapped these functions to checklist items within current reporting and best practice guidelines.

**Conclusion:**

BUGSnet is a new R package that can be used to conduct a Bayesian NMA and produce all of the necessary output needed to satisfy current scientific and regulatory standards. We hope that this software will help to improve the conduct and reporting of NMAs.

## Background

Indirect treatment comparisons (ITC) and network meta-analysis (NMA) are approaches for quantitatively summarizing an evidence base in which there are more than two treatments of interest. Unlike traditional pairwise meta-analysis, ITC/NMA can incorporate indirect evidence that arises when a group of studies evaluating different treatments share a common comparator. The incorporation of such evidence within an NMA has several advantages over pairwise meta-analysis [1, 2]. Unlike pairwise meta-analysis, an NMA allows for the comparison of two or more treatments that have never been directly compared provided that the studies examining such treatments are linked via a common comparator (i.e. an indirect comparison) [1, 2]. Another important advantage of NMA over pairwise meta-analysis is that it may provide greater statistical precision through its incorporation of indirect evidence which is not taken into account within pairwise meta-analysis [1, 2]. Lastly, an NMA can be used to rank a set of treatments for a given disease indication with respect to their clinically efficacy or harm and can be used to quantify the uncertainty surrounding such which is useful when determining policies, guidelines, and costs surrounding the choice of treatment [2].

The number of publications using NMA has increased dramatically within the past decade [3]. Despite this increase, several reviews have noted shortcomings with respect to the quality of the conduct and reporting of NMAs [4–9]. In particular, several authors have noted that a considerable proportion of NMAs do not provide a descriptive overview of the network or its structure, fail to adequately describe the statistical methods employed and whether or not their underlying assumptions were assessed and met, and lack a comprehensive summary of the results including effect estimates and measures of uncertainty regarding treatment ranks [4–9]. To improve the conduct, reporting, and appraisal of NMAs, a number of guidelines have been published which include the International Society of Pharmacoeconomics and Outcomes – Academy of Managed Care Pharmacy – National Pharmaceutical Council (ISPOR-AMCP-NPC) questionnaire for assessing the relevance and credibility of an NMA [10], the Preferred Reporting Items for Systematic Reviews and Meta-Analyses (PRISMA) extension for reporting systematic reviews incorporating NMAs of health care interventions [11], and the National Institute for Health and Care Excellence Decision Support Unit (NICE-DSU) reviewer’s checklist for appraising the synthesis of evidence within a submission to a health technology assessment agency (technical support document 7) [12].

Although the dissemination and uptake of such guidelines will hopefully help to address some of the foregoing issues, we suspect that the such issues may, in part, be related to limitations in current user-friendly software and tools used to conduct NMA. As previously noted, current software packages do not readily produce all of the output necessary to satisfy current reporting guidelines in a format that is suitable for submission to a journal or health technology assessment agency [13, 14]. Individuals must therefore rely upon multiple software packages, modify existing software, or generate code de novo in order to adhere to scientific and regulatory standards [14]. The resulting increase in time, effort, and expertise has likely impacted the quality and reporting of NMAs done to date. Furthermore, we have found that the documentation and help files of current software packages sometimes suffer from a lack of clarity regarding their implementation and use. In addition, the current lack of approachable tutorials that demonstrate how to use current NMA software could be a hindrance to users with limited programming expertise. To address these limitations, we have developed an R package called “BUGSnet” (**B**ayesian inference **U**sing **G**ibbs **S**ampling to conduct a **Net**work meta-analysis) aimed at improving the reporting and conduct of NMA/ITC. BUGSnet improves over its two main competing software packages for conducting a contrast-based Bayesian NMA: GeMTC [15] and NetMetaXL [16]. While NetMetaXL does produce much of the output necessary to satisfy reporting guidelines, it is limited in the types of analyses it can carry out. Specifically, one cannot use NetMetaXL to analyze outcomes that are not dichotomous, to conduct meta-regression, or to analyzing evidence bases with more than 15 treatments [16]. While GeMTC provides an enhanced suite of functions for conducting NMA relative to NetMetaXL, its reporting capabilities are limited. For example, GeMTC does not readily produce key reporting items for an NMA such as tabular overview of the evidence base or a SUCRA plot and league table of the NMA results on the original scale.

### Implementation

BUGSnet is a suite of functions that will carry out a Bayesian NMA while generating all items needed to satisfy the *statistical* components of the PRISMA, ISPOR-AMCP-NPC, and NICE-DSU checklists in a format that is suitable for publication or submission to a decision-making organization. These statistical components can be broadly categorized into: description of network (graphical and tabular), detection of heterogeneity, network meta-analysis (including meta-regression), model assessment, detection of inconsistency and reporting of the results. An overview of BUGSnet’s functions and the corresponding checklist items that they address is presented in Table 1.

**Table 1:** List of functions within the BUGSnet package and corresponding items on guidelines that they address

Domain	Function	Brief description	Checklist Item No.		
			PRISMA [11]	ISPOR-AMPC-NCA [10]	NICE-DSU [12]
Data preparation	data.prep()	Prepares data for further processing	N/A	N/A	N/A
Description of network	net.tab()	Descriptive statistics of evidence network	S4, 20	2, 14	A9.1, C3.1
	net.plot()	Plot of evidence network	S3	2, 14	A9.1, C3.1, C4.1
Homogeneity assessment	data.plot()	Graph of characteristics by study or treatment	18	5, 6	A7.2, B2.5, C4.2
	pma()	Heterogeneity statistics for all direct comparisons	-	5, 6	B2.1, B4.1, C4.2
Network meta-analysis	nma.model()	Specifies the NMA model (including meta-regression)	23	7, 9 10, 12, 13, 19	A6.3, A7.2, B2.3, B3.1, C2.1
	nma.run()	Runs the NMA	N/A	N/A	N/A
Model assessment	nma.diag()	Trace plots and other convergence diagnostics	–	–	B1.1
	nma.fit()	Leverage plots and deviance information criterion (DIC) values	23, S5	8, 11, 12	B2.2
Output results	nma.forest()	A forest plot of the NMA results	21	17	B4.1
	nma.league()	League table of the NMA results	21	17	B4.1
	nma.rank()	Tabular and graphical results for treatment ranking including Surface under the cumulative ranking curve (SUCRA) plot	21	18	B4.1
	nma.regplot()	Meta-regression only. Plot of estimated relative treatment effects (on the linear scale) as a function of covariate values	23	10, 13, 19	A6.3, A7.2, B2.3
	pma()	Results from pairwise comparisons	20	16	B4.1
Consistency assessment	nma.compare()	Comparison of consistency and inconsistency models [17]	S5	8	C4.3

BUGSnet is implemented within R software. BUGSnet requires that the user have installed Just Another Gibbs Sampler (JAGS) on their computer [18, 19]. Information as to how to install JAGS can be found at the program’s sourceforge homepage: http://mcmc-jags.sourceforge.net/. BUGSnet is hosted and can be accessed at the following URL: https://bugsnetsoftware.github.io/. We encourage users to submit feedback on existing code and to provide suggestions for additional functions that should be added to BUGSnet at the aforementioned homepage. Detailed vignettes describing the step-by-step use of BUGSnet to conduct an NMA on various types of outcomes are currently available in the R package documentation and on the BUGSnet homepage and additional applied examples are forthcoming.

### Data preparation

The first step to using BUGSnet is to process the data using the data.prep() function where the user specifies the name of the columns variables that correspond to the study IDs and treatment arms. This way, the user does not have to enter this information over and over in subsequent functions.

### Description of network

Current guidelines recommend that authors report plot of the evidence network [10–12]. The net.plot() and the net.tab() functions allow the user to describe the network of studies in a graphical and tabular format respectively.

With respect to the network graph, the size of the nodes and edges within the network plot are scaled such that they reflect the number of studies examining a specific treatment and the number of comparisons between any two given treatments respectively as per current recommendations. In addition, we have introduced an option that allows the user to highlight specific interventions of interest within the network graph and to label the edges with the names of the studies that have investigated these particular treatments. The colour, size, and layout of the network graph is highly customizable to ensure that the resulting figure will meet industry and journal standards.

The net.tab() function produces descriptive tables that are based on the tables produced by NetMetaXL – an excel-based software for conducting Bayesian NMAs [16]. While the tables produced by NetMetaXl are excellent descriptors of the network geometry, this software is currently only capable of handling dichotomous outcomes and is limited to 15 treatments [16]. We have expanded upon the tabular reporting of NetMetaXL by allowing such tables to summarize other types of outcomes including continuous, dichotomous, and count outcomes. An additional feature of our function is a report on whether the network is connected or not.

### Homogeneity

Current guidelines recommend a careful exploration of heterogeneity within the network, typically prior to conducting the NMA [10–12]. Researchers should identify which characteristics are likely to be important modifiers of the treatment effects a priori using content expertise or a literature review [20]. Once identified, one can use the data.plot() function within BUGSnet to assess the heterogeneity of these modifiers within an evidence network. Specifically, this function generates a graph that allows the user to display a characteristic of interest within each treatment arm, grouped by study ID or treatment.

In addition, BUGSnet also provides an option within the pma() function to produce a table summarizing a Cochrane chi-square test, the tau-squared statistic, and the I-squared statistic for assessing between-study heterogeneity within each possible pairwise comparison within the network in which there is direct evidence [21].

### Network meta-analysis

BUGSnet implements a Bayesian contrast-based NMA using a generalised linear model as described in the NICE-DSU technical support document 2 [17]. The BUGS code used to generate these models within the BUGSnet package borrows heavily from this source [17]. Within BUGSnet, the nma.model() function is used to generate the BUGS model that one wishes to fit which includes aspects such as the link function and the likelihood distribution appropriate for the outcome of interest, the choice of using a fixed effects or a random effects model, and the inclusion of covariates if one wishes to conduct a meta-regression. After the NMA model has been generated, one can run a Bayesian network meta-analysis with the function nma.run(). In the nma.run() function, the user can specify the number of burn-ins, iterations, and adaptations for the Markov Chain Monte Carlo (MCMC) algorithm and which variables they wish to monitor.

#### Bayesian inference

BUGSnet conducts NMA using Bayesian inference. There were several practical and theoretical reasons for choosing to implement the package within a Bayesian as opposed to a frequentist framework as noted by others: 1) Bayesian methods are more popular among researchers who conduct network meta-analyses; 2) Bayesian methods for network meta-analysis have been developed to a further degree; 3) Bayesian methods allow one to better handle data from trials with multiple arms and trials in which there are arms with zero events; 4) Bayesian methods are currently better suited for modeling uncertainty surrounding the heterogeneity between studies; 5) Bayesian methods present results as probabilities and are thus more suitable for ranking treatment efficacy and for incorporation into health-economic decision modeling [1, 22].

#### NMA models

BUGSnet can handle continuous, dichotomous, and count data (with or without varying follow-up times) as well as data from studies with more than two treatment arms. In what follows, we describe the NMA models that are implemented within BUGSnet. Suppose that we have data from studies *i* = 1, …, *M*. In arm *k* of study *i*, treatment *t*_*ik*_ ∈ {1, …, *T*} was used. The set {1, …, *T*} represents the set of treatments that were assessed across the *M* studies, where treatment 1 is a reference treatment. Let *a*_1_, …, *a*_*M*_ represent the number of arms in studies 1, …, *M*. Let *R*_*ik*_ be the measured aggregate response in arm *k* of study *i* (e.g. proportion of individuals who were alive at one-year, average blood pressure, etc.). Those responses are modeled as conditionally independent using an appropriate distribution *F* which is chosen based on the type of outcome at hand. For continuous outcomes, where the aggregate responses take the from of the sample mean and standard error in each arm, the distribution *F* is the normal distribution; ${R}_{ik}\sim Normal\left({\varphi}_{ik},{se}_{ik}^2\ \right)$, where *φ*_*ik*_ is the mean and ${se}_{ik}^2$ is the observed standard error of the responses in arm *k* of study *i*. When outcome is dichotomous, the distribution *F* is the binomial distribution; *R*_*ik*_~*Binomial*(*n*_*ik*_, *φ*_*ik*_ ), where *φ*_*ik*_ is the probability of experiencing the event and *n*_*ik*_ is the sample size in arm *k* of study *i*. When outcomes take the form of counts and the event rates can be assumed to be constant over the duration of follow-up, one can use the Poisson distribution; *R*_*ik*_**~***Poisson*(*e*_*ik*_*φ*_*ik*_ ), where *e*_*ik*_ is the observed person-time at risk and *φ*_*ik*_ is the event rate in arm *k* of study *i*. The latent parameters *φ*_*ik*_ ’s are transformed using an appropriate link function *g*(·) so *g*(*φ*_*ik*_) ≡ *θ*_*ik*_ can be modeled with a linear model. Table 2 summarizes the link functions *g*(·) and family distributions *F* implemented within BUGSnet based on the type of outcome data. Following the NICE-DSU technical support document 2 [17], the linear model used is generally of the contrast-based form:

$$ {\theta}_{ik}={\mu}_i+{\delta}_{ik}, $$

where *μ*_*i*_ represents the fixed effect of the treatment from arm 1 in study *i* (a control treatment) and *δ*_*ik*_ represents the (fixed or random) effect of the treatment from arm *k* of study *i* relative to the treatment in arm 1 and *δ*_*i*1_ = 0 for *i* = 1, …,*M.* In BUGSnet, two exceptions to this model occur. First, when exploring a dichotomous outcome from studies with differing lengths of follow-up time, one can use a binomial family distribution with the complementary log-log link and the linear model includes the observed follow-up time *f*_*i*_ in trial *i*: *θ*_*ik*_ = log(*f*_*i*_) + *μ*_*i*_ + *δ*_*ik*_ [17]. Second, when exploring a dichotomous outcome with a binomial family distribution and a log link, the linear model takes the form *θ*_*ik*_ = min(*μ*_*i*_ + *δ*_*ik*_, −10^−16^) to ensure that *θ*_*ik*_ is negative and the probabilities *φ*_*ik*_ are between 0 and 1.

**Table 2:** Types of outcomes and corresponding link functions and likelihood distributions available within BUGSnet

Type of Outcome	Arm-Level Data Required	Distribution Family	Link function	Measure of effect	Assumption on follow-up time
Continuous	Mean & Standard Error	Normal	Identity	Mean Difference	Outcome is unrelated to follow-up time
Dichotomous	Events & Sample Size	Binomial	Logit	Odds Ratio	
			Log	Risk Ratio	
	Events & Sample Size & Median Follow-Up Time		Complementary log-log	Hazard Ratio	Event rates are constant over the duration of follow-up
Count	Events & Person-Time at Risk	Poisson	Log	Rate Ratio	

In a random effect model, the ${\boldsymbol{\delta}}_i'\mathrm{s}={\left({\delta}_{i2},\dots, {\delta}_{i{a}_i}\right)}^{\top }$ are modeled as conditionally independent with distributions

1 $$ \left[{\boldsymbol{\delta}}_i|{\mathbf{d}}_i,\varSigma \right]\sim MVNormal\left({\mathbf{d}}_i,\varSigma \right), $$

where ${\mathbf{d}}_i={\left({d}_{\left({t}_{i1},{t}_{i2}\right)},\dots, {d}_{\left({t}_{i1},{t}_{i{a}_i}\right)}\right)}^{\top }$ and ${d}_{\left({t}_{i1},{t}_{ik}\right)}={d}_{\left(1,{t}_{ik}\right)}-{d}_{\left(1,{t}_{i1}\right)}$ is the difference in the treatment effect of treatments *t*_*i*1_ and *t*_*ik*_ on the *g*(·) scale and *d*_(1, 1)_ = 0. For *Σ* we adopt the usual compound symmetry structure described in (16), with variances *σ*^2^ and covariances 0.5*σ*^2^, where *σ*^2^ represents the between-trial variability in treatment effects (heterogeneity). Independent priors are used on *σ*, *d*_(1, 2)_, …. ,*d*_(1, *T*)_ and *μ*_1_, …, *μ*_*M*_. For ease of implementation, in BUGSnet, the distribution (1) is decomposed into a series of conditional distributions [17].

$$ \left[{\delta}_{ik}|{\delta}_{i2},\dots, {\delta}_{ik-1},{\mathbf{d}}_i,\varSigma \right]\sim Normal\left({d}_{\left({t}_{i1},{t}_{ik}\right)}+\frac{1}{k-1}{\sum}_{j=1}^{k-1}\left[{\delta}_{\mathrm{ij}}-{d}_{\left({t}_{i1},{t}_{ik}\right)}\right],\frac{k}{2\left(k-1\right)}{\sigma}^2\right). $$

In a fixed effect model, the *δ*_*ik*_ ’ s are treated as “fixed” (to use frequentist jargon) and are defined as ${\delta}_{ik}={d}_{\left({t}_{i1},{t}_{ik}\right)}={d}_{\left(1,{t}_{ik}\right)}-{d}_{\left(1,{t}_{i1}\right)}$ with *d*_(1, 1)_ = 0. Independent priors are used on *d*_(1, 2)_, …. ,*d*_(1, *T*)_ and *μ*_1_, …, *μ*_*M*_. In both the fixed and random-effects model, the posterior quantities of interest are all the mean treatment contrasts ${d}_{\left({t}_{i1},{t}_{ik}\right)}$ which can be determined from *d*_(1, 2)_, …. ,*d*_(1, *T*)_ through the transitivity relation ${d}_{\left({t}_{i1},{t}_{ik}\right)}={d}_{\left(1,{t}_{ik}\right)}-{d}_{\left(1,{t}_{i1}\right)}.$

#### Meta-regression

Let *x*_*ik*_ be a continuous covariate available in arms *k* = 1, …, *a*_*i*_ of studies *i* = 1, …, *M*. Network meta-regression is implemented in BUGSnet via the linear model

$$ {\theta}_{ik}={\mu}_i+{\delta}_{ik}+{\beta}_{\left({t}_{i1},{t}_{ik}\right)}\left({x}_{ik}-\overline{x}\right), $$

where $\overline{x}$ is the average of the *x*_*ik*_ ’s across studies and the ${\beta}_{\left({t}_{i1},{t}_{ik}\right)}={\beta}_{\left(1,{t}_{ik}\right)}-{\beta}_{\left(1,{t}_{i1}\right)}$ are regression coefficients for the effect of the covariate on the relative effect of treatments *t*_*i1*_ and *t*_*ik*_, with *β*_(1, 1)_ = … = *β*_(*T*, *T*)_ = 0. A prior is used on *β*_(1, 2)_, …, *β*_(1, *K*)_. When conducting a meta-regression analysis, the output plots and tables described in the Output section (league heat plot, league table, etc.) can also be produced but the user will need to specify a value for the covariate at which to produce treatment comparisons. Those treatment comparisons are calculated internally within BUGSnet by computing posterior quantities of interest at a specific covariate value *x*^0^ as ${d}_{\left({t}_{i1},{t}_{ik}\right)}+{\beta}_{\left({t}_{i1},{t}_{ik}\right)}\left({x}^0-\overline{x}\right),$ and using the transitivity relations ${d}_{\left({t}_{i1},{t}_{ik}\right)}={d}_{\left(1,{t}_{ik}\right)}-{d}_{\left(1,{t}_{i1}\right)}$ and ${\beta}_{\left({t}_{i1},{t}_{ik}\right)}={\beta}_{\left(1,{t}_{ik}\right)}-{\beta}_{\left(1,{t}_{i1}\right)}.$

#### Choice of priors

By default, BUGSnet implements the vague priors described in Table 3. Our choice of priors was based on the justifications made by van Valkenhoef et al. (2012) [15] which allow a prior variance to be easily calculated from the data without any user input. These priors are the same as the ones implemented in the GeMTC R package [15]. The user also has the option within the nma.model() function to specify their own prior which is useful for conducting sensitivity analyses, namely for the comparison of prior distributions on the random effects standard deviation, *σ*, to insure that they do not have a significant effect on the posterior estimates.

**Table 3:** Priors implemented by default in BUGSnet

	Consistency Model		Inconsistency Model	
Parameters	Random effect	Fixed effect	Random effect	Fixed effect
*μ*_1_, …, *μ*_*M*_	iid N(0,(15*u*)^2^) Except when a log link is used with a binomial family, in which case *μ*_*i*_ = log(*p*_*i*_), *p*_*i*_~ iid U(0,1) as per Warn et al. [23]			
*d*_1, 2_, …. ,*d*_1, *T*_	iid N(0,(15*u*)^2^)		NA	
*d*_1, 2_, …. ,*d*_1, *T*_, …, *d*_*T* − 2, *T* − 1_, *d*_*T* − 2, *T*_, *d*_*T* − 1, *T*_	NA		iid N(0,(15*u*)^2^)	
*σ*	U(0,*u*)	NA	U(0,*u*)	NA
*β*_(1, 2)_, …, *β*_(1, *K*)_ (meta-regression only)	Unrelated: iid t(0, *u*^2^, df = 1) Exchangeable: iid N(*b*, *γ*^2^), *b*~ t(0, *u*^2^, df = 1), *γ*~*U*(0, *u*) Equal: *β*_2_ = … = *β*_*T*_ = *B*, *B*~ t(0, *u*^2^, df = 1)			

The variances 15*u* are taken from van Valkenhoef (2012) et al., where *u* is the largest maximum likelihood estimator of treatment differences on the linear scale in single trials [15]. Note that t denotes Student’s t distribution with parameters: location, variance and degrees of freedom.

### Model assessment

After the NMA model has been run, guidelines recommend that one assesses the convergence and fit of the model [10–12]. In BUGSnet, convergence can be assessed using trace plots and other convergence diagnostics produced by the nma.diag() function. Lastly, the fit of the model and the identification of potential outliers can be carried out using the nma.fit() function which will produce a plot of the leverage values and also display the corresponding effective number of parameters, total residual deviance, and deviance information criterion (DIC). These latter values can be used to help determine or justify model choice when considering two or more competing models (e.g. between a fixed- or random-effects model) and to help identify data points that contribute heavily to the DIC and/or that are influential.

### Consistency

A fundamental assumption of an NMA is the assumption of transitivity [2]. Under this assumption, one assumes that one can estimate the difference in the effect of two treatments by subtracting the difference in the effects of the two treatments relative to a common comparator as follows: ${d}_{\left({t}_{i1},{t}_{ik}\right)}={d}_{\left(1,{t}_{ik}\right)}-{d}_{\left(1,{t}_{i1}\right)}$[2]. Aside from exploring clinical heterogeneity of treatment definitions and modifiers within the network using the data.plot() function, one can also detect violations of the assumption of transitivity by examining statistical consistency within the network. Statistical consistency refers to the statistical agreement between indirect and direct evidence within an evidence network [2]. Evidence of inconsistency would indicate a violation of the transitivity assumption. As noted by Efthimiou et al. (2015), statistical consistency can only be explored if there are closed loops within the network [2]. A variety of methods have been proposed to assess consistency within a network meta-analysis [2, 24, 25]. Such methods are often categorized as being “global” or “local” depending upon whether they examine inconsistency within the entire network or within particular segments thereof [2]. BUGSnet currently implements the inconsistency model (or unrelated mean effects model) as described in the NICE-DSU TSD 4 [26]. An inconsistency model is an NMA model similar to the consistency models described above but transitivity ${d}_{\left({t}_{i1},{t}_{ik}\right)}={d}_{\left(1,{t}_{ik}\right)}-{d}_{\left(1,{t}_{i1}\right)}$ is not assumed. Instead, independent priors are defined on each of the ${d}_{\left({t}_{i1},{t}_{ik}\right)}$ ’s. Inconsistency models therefore have more parameters than consistency models, which needs to be weighted against how well they fit the data compared to the consistency model to determine if there is evidence of inconsistency. The inconsistency model can be specified using the type = "inconsistency" option in the nma.model(). To examine inconsistency at the global level, the fit of the inconsistency model can be compared against a model in which consistency is assumed using the nma.fit() function and comparing the DICs. Local inconsistency can be explored on the leverage plots produced by nma.fit() and also using the nma.compare() function which produces a plot comparing the posterior mean deviance of each data point between the consistency and the inconsistency model.

We chose to implement the inconsistency model method for assessing inconsistency in BUGSnet because it easily handles different network structures and multi-arm trials, which is not the case with other methods for assessing inconsistency such as the Bucher method [26, 27]. More options for assessing inconsistency at both the global and local levels will be considered in further BUGSnet releases.

### Output

We provide several functions for displaying the results of the NMA in both graphical and tabular formats (league tables, league heat plots, SUCRA plots, SUCRA tables, rankograms and forest plots) to satisfy current guidelines. With respect to plotting the magnitude and uncertainty of the treatment effects, users can the use the nma.forest() function to graph the effect estimates from the NMA against a comparator specified by the user. The effect estimates can also be presented within a league table using the nma.league() function. An important presentation feature in BUGSnet, particularly for large league tables, is that the user can specify an option to colour and arrange the league table into a heatmap that highlights the magnitude of the effect estimates. Users can also graphically display the probability of the ranking of each treatment within a surface under the cumulative ranking curve (SUCRA) plot which can be specified within the nma.rank() function. This function can also be used to present treatment ranks in a tabular format, extract SUCRA values and produce a rankogram. All of the plots produced by these three reporting functions are produced with the ggplot2 package. As such, the user can easily customize the plots (e.g. change the background, add a title) by adding layers using the + command. Also, for reporting relative treatment effects the user can specify whether they want to plot the results on the the linear scale (log scale) or the original scale.

When meta-regression is conducted, the nma.rank(), nma.forest() and nma.league() functions allow the user to specify for which value of the covariate they wish to present the results. Even though the covariate is centered for meta-regression, the user does not have to do any conversion and results are provided on the original non-centered scale. Another function, nma.regplot() outputs a plot of relative treatment effects on the linear scale across the range of covariate values used in the meta-regression, as in the NICE-DSU TSD 3 [28].

It is sometimes recommended that users present results from the direct evidence where available [29]. To accommodate this, we have also incorporated the pma() function within BUGSnet which will perform pairwise meta-analysis using the meta package in R and automatically output the results into a tabular format [30].

## Results

The following is a demonstration of some of the functions contained within BUGSnet (Table 1) and some of the possible outputs. To accomplish this task, we have recreated an analysis of a dichotomous outcome where studies had variable follow-up times described in the NICE-DSU technical support document 2 (referred to as “Data Example 3”) [17]. The BUGSnet code used to produce this analysis is available in the vignette titled *survival* in the BUGSnet documentation, and appended as a supplement to this article (see Additional file 1). Additional outputs are presented in the vignette as well as a more detailed description of how to conduct and report network meta-analysis, which is only presented here in brief.

The evidence network used in this analysis consists of 22 randomized trials (including multi-arm trials) that examined the effects of six antihypertensive treatments on the risk of developing diabetes [31]. The outcome for this data is the number of new diabetes cases observed during the trial period. The data is organized in the long format (i.e. one row per treatment arm), with variables indicating the study ID, the treatment ID, the number of patients, the number of events, and the mean age (and standard deviation) of participants for each treatment arm (see Table 4). The results of our package are concordant with those reported in the TSD as well results obtained with GeMTC (code and outputs provided as supplement to this article (see Additional files 2, 3, 4 and 5) and NetMetaXL.

**Table 4:** Organization of diabetes dataset used to demonstrate the capabilities of BUGSnet

Study ID	Treatment	Number of Participants	Number of Events	Age (Mean)	Age (SD)
MRC-E	Diuretic	1081	43	60.7	14.3
MRC-E	Placebo	2213	34	59.2	13.1
MRC-E	blocker	1102	37	60.2	14.0
EWPH	Diuretic	416	29	59.0	15.2
EWPH	Placebo	424	20	57.0	14.8
.					
.					
.					
VALUE	CCB	5074	845	56.4	13.1
VALUE	ARB	5087	690	57.8	13.0

### Data preparation, description of network and homogeneity

**Fig. 1:**
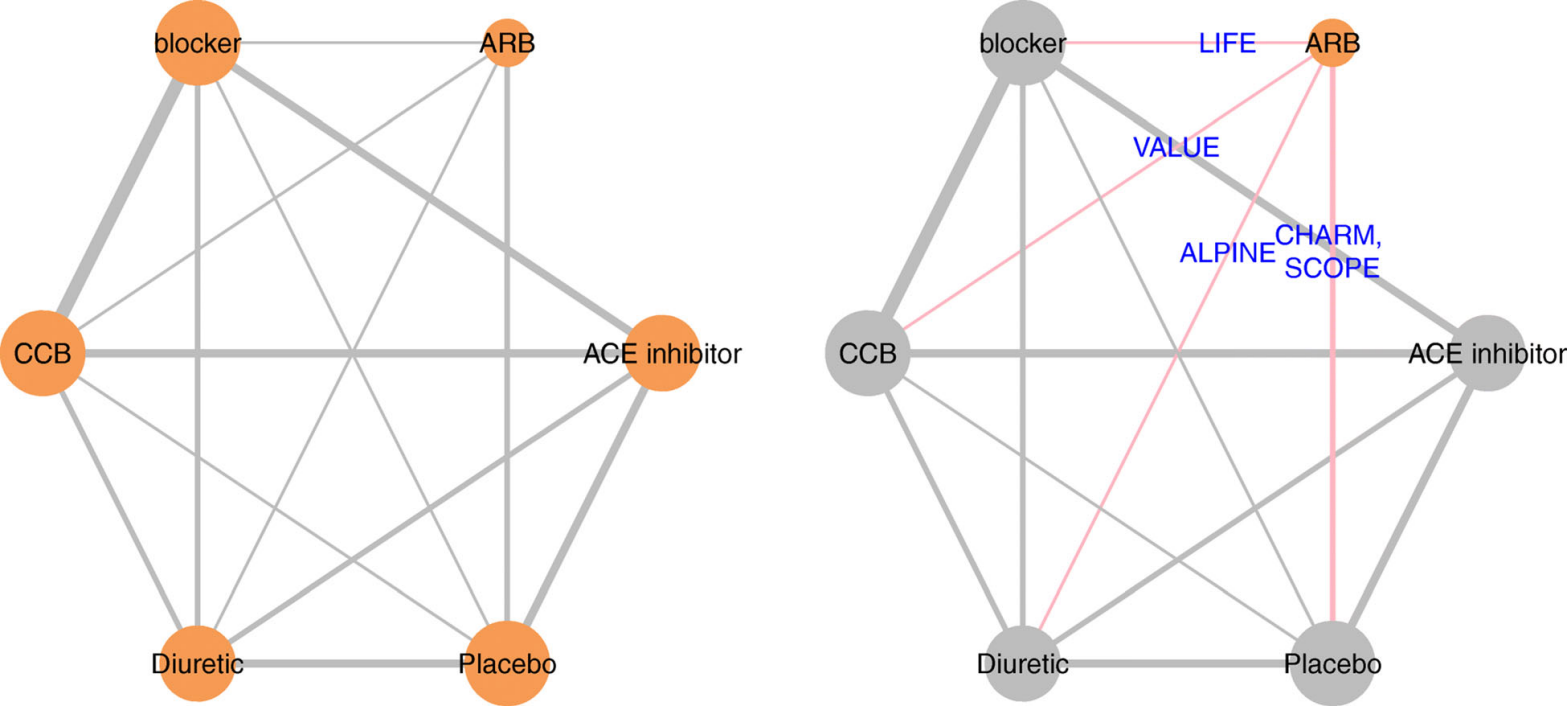
Network plots produced by the net.plot() Function in BUGSnet

**Fig. 2:**
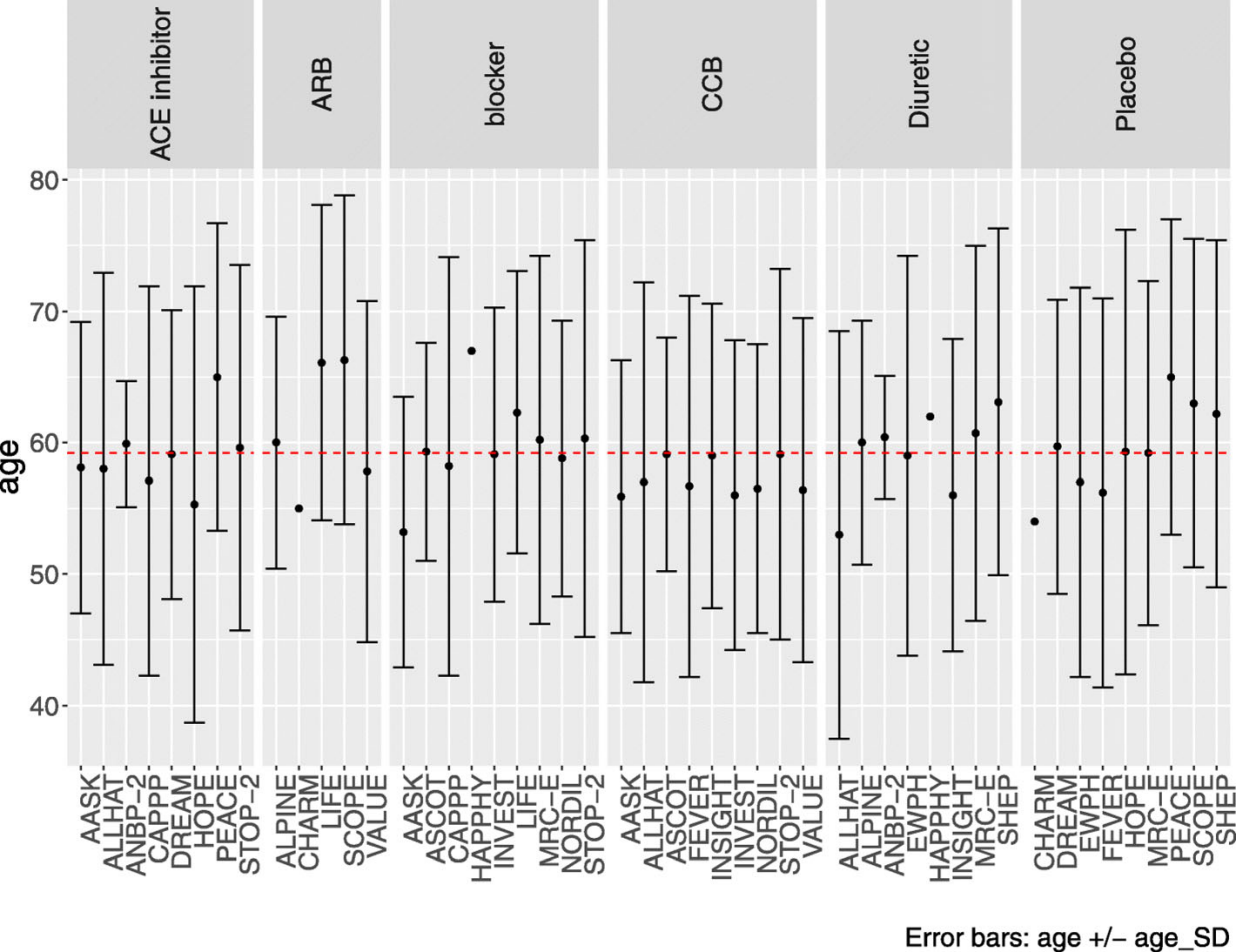
Graph of patient characteristic by treatment using the data.plot() function in BUGSnet

After the data was prepared using the data.prep() function, the net.plot() and the net.tab() functions were used to describe the network of studies in a graphical (Fig. 1) and tabular format respectively (Table 5). As previously discussed, the assumptions of network meta-analysis will be violated when an effect modifier is heterogeneously distributed throughout an evidence base [20]. Prior to conducting the network meta-analysis, analysts can use the data.plot() function to examine the distribution of an effect modifier within the network. The determination of whether or not a variable is an effect modifier and whether or not the observed differences in its distribution are clinically meaningful is determined according to expert opinion and prior evidence. To demonstrate this function, we have simulated a patient characteristic that may modify the treatment effect (i.e. the age of participants). To mimic a lack of reporting, we have omitted the standard deviation for a few of the studies. As observed in Fig. 2, the mean age of participants within each treatment arm (the individual points) is similar to the overall mean age of participants within the evidence base (the red dotted line). According to the standard deviation (the +/− error bars), the variability of ages within each treatment arm appear to be similar as well (where available). Based on this analysis, one would conclude that there is no meaningful heterogeneity in the distribution of age. This analysis would be repeated for all potentially important effect modifiers identified a priori by clinical opinion and a review of previous studies. If no heterogeneity is detected, then one may proceed to conducting the network meta-analysis. If heterogeneity is detected, one can attempt to adjust for imbalances by using meta-regression (if there are an adequate number of studies) or by using alternative statistical techniques that leverage individual patient data (e.g. matching-adjusted indirect comparison or simulated treatment comparison) [20].

**Table 5:** Network characteristics produced by the net.tab() function in BUGSnet

Characteristic	Value
Number of Interventions	6
Number of Studies	22
Total Number of Patients in Network	154,176
Total Possible Pairwise Comparisons	15
Total Number of Pairwise Comparisons With Direct Data	14
Is the network connected?	TRUE
Number of Two-arm Studies	18
Number of Multi-Arms Studies	4
Total Number of Events in Network	10,962
Number of Studies With No Zero Events	22
Number of Studies With At Least One Zero Event	0
Number of Studies with All Zero Events	0
Mean person follow up time	4.06

### Network meta-analysis

**Fig. 3:**
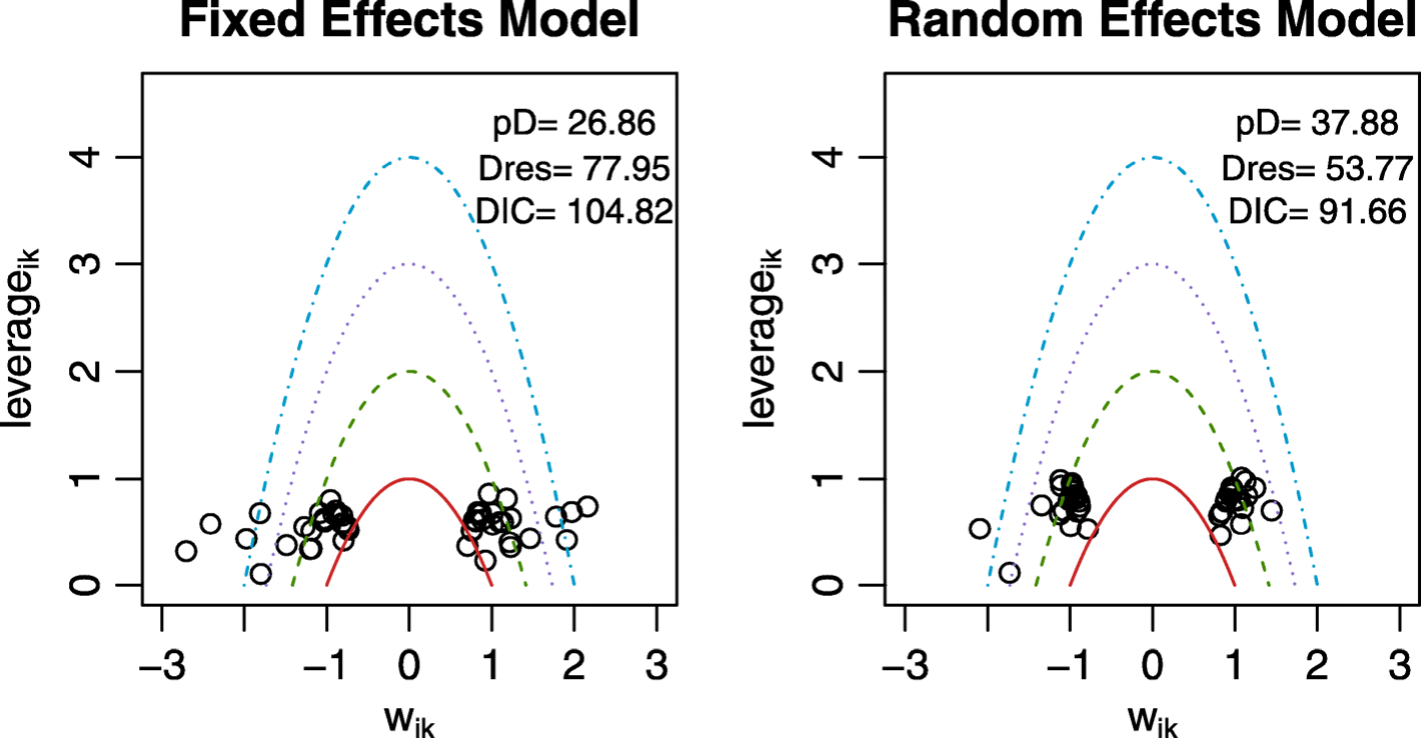
Leverage plots and fit statistics produced by the nma.fit() Function in BUGSnet

We conducted an NMA on the Diabetes dataset by fitting a generalized linear model with a complementary log-log link function and binomial likelihood function to account for the dichotomous outcome and differing follow-up times between studies, which was specified through the use of nma.model(). To be consistent with the NICE-DSU technical support document, we specified a burn-in of 50,000 iterations followed by 100,000 iterations with 10,000 adaptations in the nma.run() function. We compared the fit of both a fixed- and random-effects model. According to a visual examination of the leverage plots and comparison of the DIC values produced by the nma.fit(), the random effects model would be preferred over the fixed effects model for this particular dataset because the DIC value is lower and because there are fewer outliers in the leverage plot (Fig. 3).

### Output

**Fig. 4:**
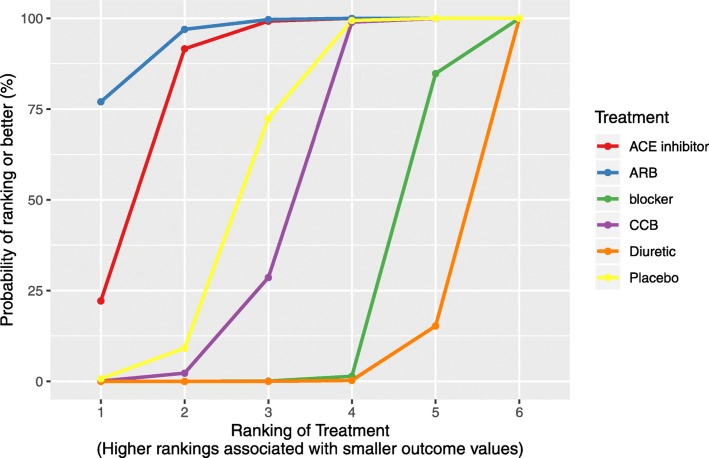
SUCRA plot produced by the nma.rank() Function in BUGSnet

**Fig. 5:**
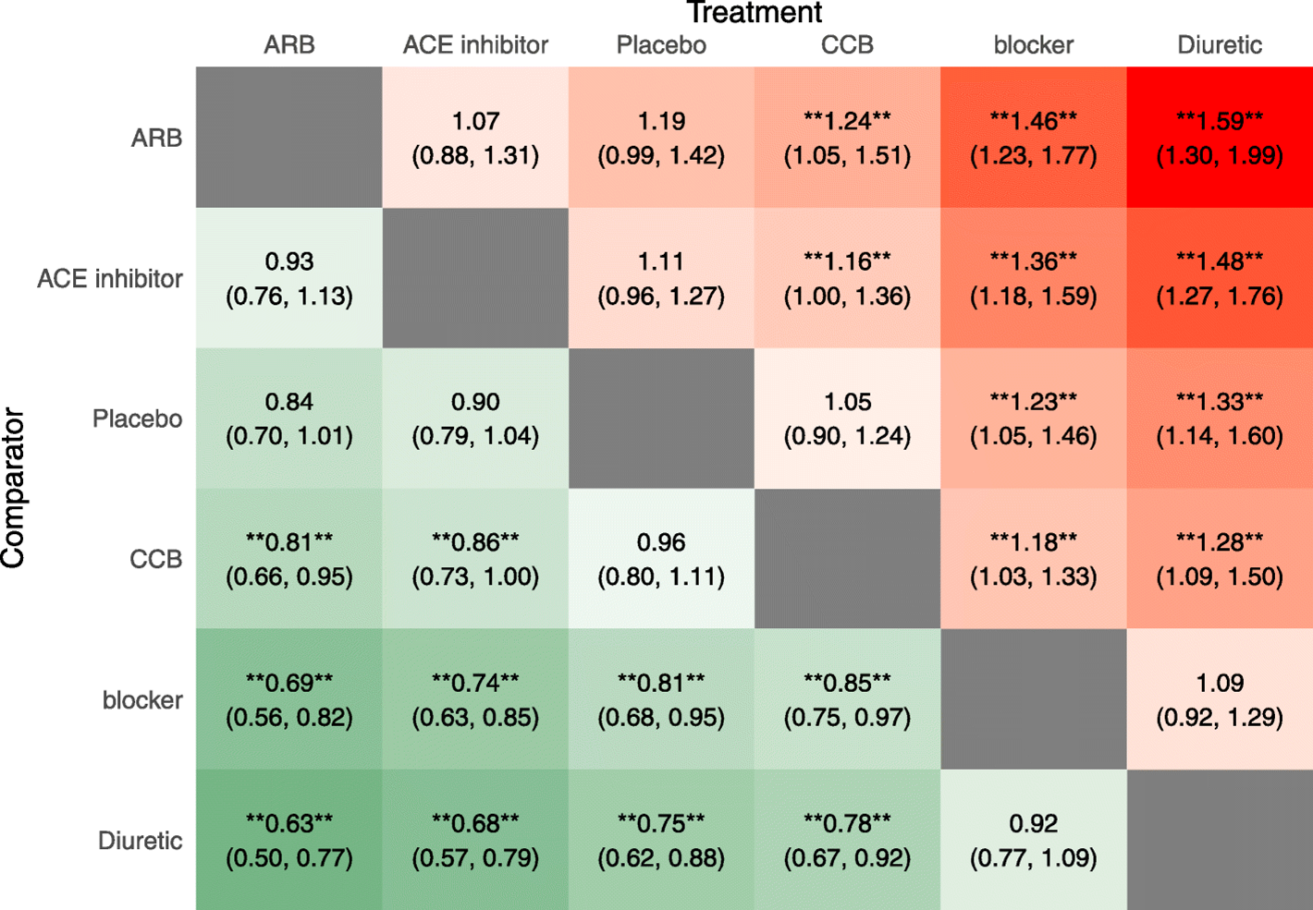
League Table Heatmap Produced by the nma.league() Function in BUGSnet. Legend: The values in each cell represent the relative treatment effect (and 95% credible intervals) of the treatment on the top, compared to the treatment on the left. A double asterisk indicates statistical significance

We present results from the generalized linear model that we previously fit to the Diabetes dataset. As visualized in the SUCRA plot obtained from nma.rank(), the angiotensin-receptor blockers’ (ARB) curve is consistently above the other treatments’ curves suggesting that it is the most beneficial treatment with respect to the outcome among the treatments included in the Diabetes evidence network (Fig. 4). The effect estimates and credible intervals produced by the foregoing model are displayed in a league heat plot (Fig. 5) obtained using nma.league(). In Fig. 5, one can see that the difference between ARB and other treatments are all statistically significant at the 95% level except for the ACE inhibitor and Placebo treatments.

### Consistency

**Fig. 6:**
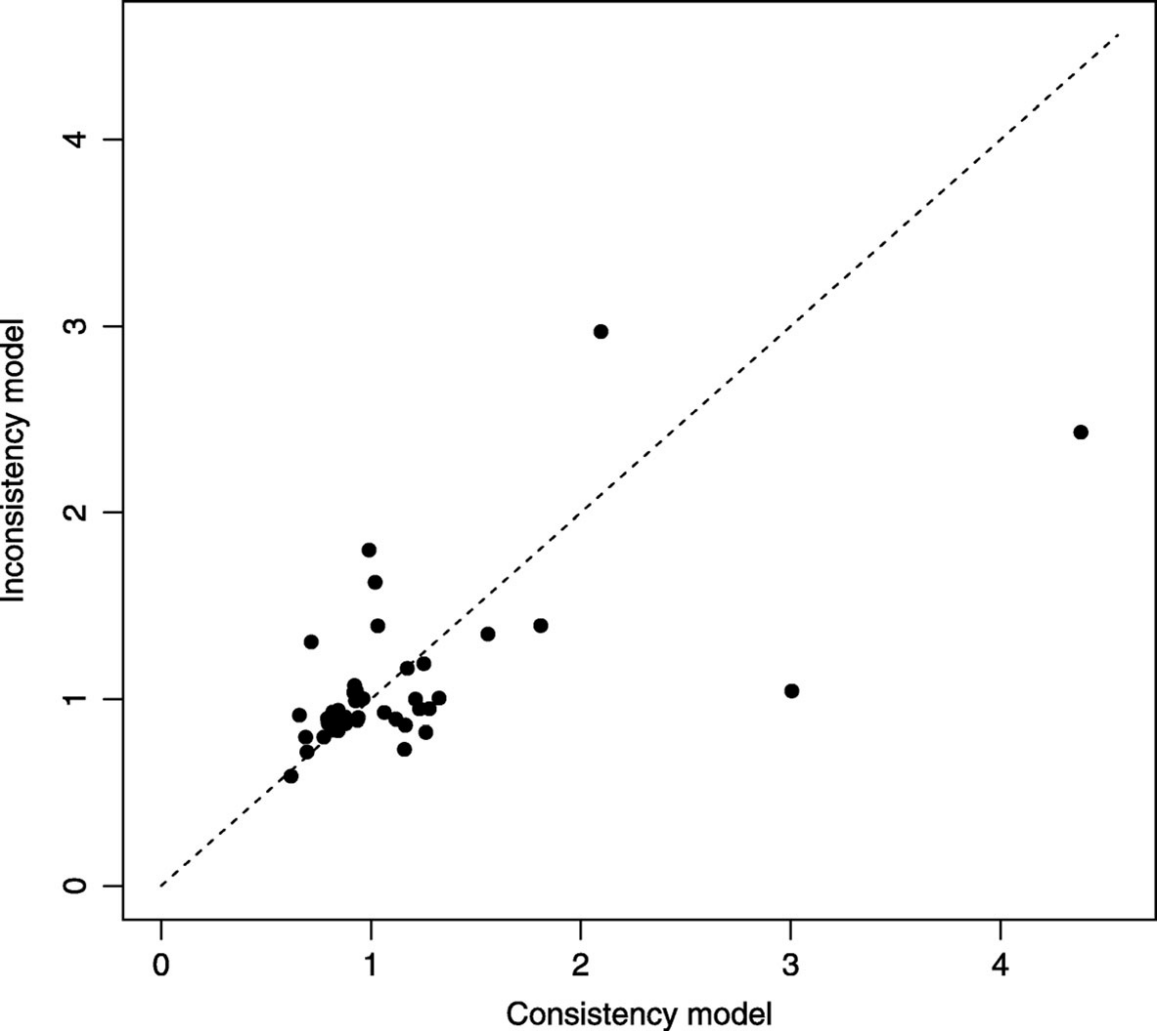
Posterior mean deviance comparison plot produced by the nma.compare() Function in BUGSnet.][Legend: Each data point represents a treatment arm’s contribution to posterior mean deviance for the consistency model (horizontal axis) and the inconsistency model (vertical axis)

To assess the presence of inconsistency, we fit an NMA model similar to the one previously described but assuming inconsistency. We obtain leverage plots similar to Fig. 3 using the nma.fit() function where we find that the DIC for the consistency model is marginally smaller than for the inconsistency mode. We also use the nma.compare() function to plot the individual data points’ posterior mean deviance contributions for the consistency model vs the inconsistency model (Fig. 6) as recommended in the NICE-DSU TSD 4 [26]. Overall, we conclude that there is a lack of evidence to suggest inconsistency within the network.

## Discussion

BUGSnet is intended to be used by researchers when assessing the clinical efficacy of multiple treatments within the context of a submission to a journal or a health technology assessment agency. For conducting a contrast-based Bayesian NMA, the two main competing software packages that one may consider are GeMTC [15] and NetMetaXL [16], for which we have discussed limitations in the introduction. With BUGSnet, we aimed to create a single tool that would compete with the reporting capabilities of NetMetaXL and the analytic capabilities of GeMTC. We have also aimed to provide users with enhanced reporting options not included in existing software such as a function to produce graphs that show the distribution of effect modifiers by trial or by treatment arm and an option to print study names and highlight certain treatment comparisons within the network plot. To help facilitate the use of BUGSnet among new users, we have provided three vignettes (with more vignettes forthcoming) in the R help files that walk users through conducting an NMA using BUGSnet by providing detailed R code and interpretations of the statistical output. Despite these benefits, there are limitations of BUGSnet. BUGSnet is currently limited to exclusively analyzing arm-level data. In contrast, GeMTC can be used to conduct an NMA using entirely arm-level or entirely contrast-level data [22]. Relative to GeMTC, another limitation of BUGSnet is that GeMTC currently provides a broader range of methods of assessing inconsistency such as the node-splitting method and a broader range of meta-regression analyses such as subgroup meta-analysis. Since it is implemented within the R environment, some users may find BUGSnet more difficult to use relative to NetMetaXL, which is implemented within Microsoft Excel. At this point, arm-based models [22] have not been implemented in BUGSnet; the R package pcnetmeta allows such analyses, although it does not readily provide a complete suite of outputs like BUGSnet. We plan to address these shortcomings in future iterations of BUGSnet and interested users should check the previously mentioned URL for updates.

Network meta-analysis is a rapidly evolving area of research with new methods constantly being developed [32]. While the work presented within this paper provides the essential tools required to conduct an NMA in accordance with current guidelines, we plan to implement additional functions and features within this package, based on user feedback, to provide enhanced flexibility and to ensure relevance. Some of the preliminary requests for short-term additions include: 1) additional functions for detecting inconsistency within the network such as the Bucher method [27]; 2) an option to allow the user to conduct an NMA using study-level effect estimates; 3) allowing for the relaxation of the proportional hazards assumption when analyzing time-to-event outcomes; 4) allowing for sub-group meta-regression and the inclusion of more than one covariate into the meta-regression model; 5) a function that will automatically generate a report or slide deck presentation of the results that could be saved as a pdf, html or Word.

As detailed in Table 1, the functions contained within BUGSnet can be used to address the items within the PRISMA, ISPOR-AMCP-NPC, and NICE-DSU reporting guidelines that are related to the statistical analysis component of an NMA [11, 12, 29]. However, it should be emphasised that there are several non-statistical issues described within these guidelines that BUGSnet is not meant to address such as the identification of the research question, the specification of the study population and competing interventions, the development of the search strategy, and the assessment of the risk of bias within each study [10–12]. Researchers are urged to consult with these guidelines when planning their NMA to ensure that all aspects of the NMA, both statistical and non-statistical, adhere to current reporting and methodologic standards.

## Conclusions

Here we present a new JAGS-based R package for conducting Bayesian NMA called BUGSnet. Relative to existing NMA software, BUGSnet provides an enhanced set of tools for conducting and reporting results according to published best-practice guidelines to help overcome the lack of quality identified within this body of literature. In addition to these features, we have attempted to provide ample documentation describing the use and implementation of BUGSnet to help promote the understanding and uptake of this software. Lastly, we plan to monitor the literature and to implement new features within BUGSnet based on the NMA analyst community to ensure that the package remains up-to-date with the latest advances in this rapidly developing area of research.

## Availability and requirements

Project name: BUGSnet

Project home page: https://bugsnetsoftware.github.io/

Operating system(s): Windows 10 v1809 and Mac OS 10.14 (may work on earlier versions but not tested)

Programming language: R

Other requirements: JAGS 4.3.0

License: Creative Commons Attribution-NonCommercial-ShareAlike 4.0 International

Any restriction to use by non-academics: Contact authors for non-academic use.

## Supplementary information

**Additional file 1.** Vignette on network meta-analysis of survival data. A vignette detailing how to obtain the outputs in the Results section using BUGSnet version 1.0.2. The vignette includes all the necessary R code as well as additional outputs and explanations that were not presented in this manuscript for the sake of brevity.

**Additional file 2.** R code to compare BUGSnet with GeMTC for the analysis of the diabetes data.

**Additional file 3.** Diabetes dataset to accompany the R code in Additional file 2.

**Additional file 4.** BUGSnet league table obtained from the R code in Additional file 2.

**Additional file 5.** GeMTC league table obtained from the R code in Additional file 2.

## Data Availability

All of the datasets and material contained within the manuscript can be accessed within the BUGSnet package via the BUGSnet homepage: https://bugsnetsoftware.github.io/

## Abbreviations

ISPOR-AMCP-NPA: International Society for Pharmacoeconomics and Outcomes Research - Academy of Managed Care Pharmacy - National Pharmaceutical Council
ITC: Indirect Treatment Comparisons
JAGS: Just Another Gibbs Sampler
NICE-DSU: National Institute for Health and Care Excellence Decision Support Unit
NMA: Network Meta-Analysis
PRISMA: Preferred Reporting Items for Systematic Reviews and Meta-Analysis
SUCRA: Surface Under the Cumulative Ranking Curve
