# Supplementary material for: BUGSnet: an R package to facilitate the conduct and reporting of Bayesian network Meta-analyses
Source: BMC Med Res Methodol. 2019 Oct 22;19:196. doi: 10.1186/s12874-019-0829-2 (PMC6805536; doi:10.1186/s12874-019-0829-2)
Supplement: Supplementary file 1 — Additional file 1. Vignette on network meta-analysis of survival data. A vignette detailing how to obtain the outputs in the Results section using BUGSnet version 1.0.2. The vignette includes all the necessary R code as well as additional outputs and explanations that were not presented in this manuscript for the sake of brevity. [file 12874_2019_829_MOESM1_ESM.html]

NMA for Survival Outcomes


# NMA for Survival Outcomes

#### Justin Slater, Audrey Beliveau

#### 2019-08-30

The purpose of this example is to demonstrate how to use BUGSnet to perform network meta-analysis on time-to-event data, where each study has a different followup time. The data was taken from the NICE DSU TSD2 (Dias et al. 2011), page 66. The results of our package are concordant with those reported in the TSD.

The Diabetes data has 22 studies and 6 treatments, each study has a follow up time between 1 and 6.1 years. The outcome for this data (`diabetes.sim`) is the number of new diabetes cases observed during the trial period. An age variable, including its standard deviation, was simulated for demonstration purposes. This data contains multi-arm trials, which can easily be accomodated using BUGSnet. The data is shown here:

| Study | followup | trtnum | Treatment | diabetes | n | age | age\_SD | age\_type |
| --- | --- | --- | --- | --- | --- | --- | --- | --- |
| MRC-E | 5.8 | 1 | Diuretic | 43 | 1081 | 60.7 | 14.26 | mean & SD |
| MRC-E | 5.8 | 2 | Placebo | 34 | 2213 | 59.2 | 13.10 | mean & SD |
| MRC-E | 5.8 | 3 | blocker | 37 | 1102 | 60.2 | 14.00 | mean & SD |
| EWPH | 4.7 | 1 | Diuretic | 29 | 416 | 59.0 | 15.20 | mean & SD |
| EWPH | 4.7 | 2 | Placebo | 20 | 424 | 57.0 | 14.80 | mean & SD |
| SHEP | 3.0 | 1 | Diuretic | 140 | 1631 | 63.1 | 13.20 | mean & SD |
| SHEP | 3.0 | 2 | Placebo | 118 | 1578 | 62.2 | 13.20 | mean & SD |
| HAPPHY | 3.8 | 1 | Diuretic | 75 | 3272 | 62.0 | NA | mean |
| HAPPHY | 3.8 | 3 | blocker | 86 | 3297 | 67.0 | NA | mean |
| ALLHAT | 4.0 | 1 | Diuretic | 302 | 6766 | 53.0 | 15.50 | mean & SD |
| ALLHAT | 4.0 | 4 | CCB | 154 | 3954 | 57.0 | 15.20 | mean & SD |
| ALLHAT | 4.0 | 5 | ACE inhibitor | 119 | 4096 | 58.0 | 14.90 | mean & SD |
| INSIGHT | 3.0 | 1 | Diuretic | 176 | 2511 | 56.0 | 11.90 | mean & SD |
| INSIGHT | 3.0 | 4 | CCB | 136 | 2508 | 59.0 | 11.60 | mean & SD |
| ANBP-2 | 4.1 | 1 | Diuretic | 200 | 2826 | 60.4 | 4.70 | mean & SD |
| ANBP-2 | 4.1 | 5 | ACE inhibitor | 138 | 2800 | 59.9 | 4.80 | mean & SD |
| ALPINE | 1.0 | 1 | Diuretic | 8 | 196 | 60.0 | 9.30 | mean & SD |
| ALPINE | 1.0 | 6 | ARB | 1 | 196 | 60.0 | 9.60 | mean & SD |
| FEVER | 3.3 | 2 | Placebo | 154 | 4870 | 56.2 | 14.80 | mean & SD |
| FEVER | 3.3 | 4 | CCB | 177 | 4841 | 56.7 | 14.50 | mean & SD |
| DREAM | 3.0 | 2 | Placebo | 489 | 2646 | 59.7 | 11.20 | mean & SD |
| DREAM | 3.0 | 5 | ACE inhibitor | 449 | 2623 | 59.1 | 11.00 | mean & SD |
| HOPE | 4.5 | 2 | Placebo | 155 | 2883 | 59.3 | 16.90 | mean & SD |
| HOPE | 4.5 | 5 | ACE inhibitor | 102 | 2837 | 55.3 | 16.60 | mean & SD |
| PEACE | 4.8 | 2 | Placebo | 399 | 3472 | 65.0 | 12.00 | mean & SD |
| PEACE | 4.8 | 5 | ACE inhibitor | 335 | 3432 | 65.0 | 11.70 | mean & SD |
| CHARM | 3.1 | 2 | Placebo | 202 | 2721 | 54.0 | NA | mean |
| CHARM | 3.1 | 6 | ARB | 163 | 2715 | 55.0 | NA | mean |
| SCOPE | 3.7 | 2 | Placebo | 115 | 2175 | 63.0 | 12.50 | mean & SD |
| SCOPE | 3.7 | 6 | ARB | 93 | 2167 | 66.3 | 12.50 | mean & SD |
| AASK | 3.8 | 3 | blocker | 70 | 405 | 53.2 | 10.30 | mean & SD |
| AASK | 3.8 | 4 | CCB | 32 | 202 | 55.9 | 10.40 | mean & SD |
| AASK | 3.8 | 5 | ACE inhibitor | 45 | 410 | 58.1 | 11.10 | mean & SD |
| STOP-2 | 4.0 | 3 | blocker | 97 | 1960 | 60.3 | 15.10 | mean & SD |
| STOP-2 | 4.0 | 4 | CCB | 95 | 1965 | 59.1 | 14.10 | mean & SD |
| STOP-2 | 4.0 | 5 | ACE inhibitor | 93 | 1970 | 59.6 | 13.90 | mean & SD |
| ASCOT | 5.5 | 3 | blocker | 799 | 7040 | 59.3 | 8.30 | mean & SD |
| ASCOT | 5.5 | 4 | CCB | 567 | 7072 | 59.1 | 8.90 | mean & SD |
| NORDIL | 4.5 | 3 | blocker | 251 | 5059 | 58.8 | 10.50 | mean & SD |
| NORDIL | 4.5 | 4 | CCB | 216 | 5095 | 56.5 | 11.00 | mean & SD |
| INVEST | 4.0 | 3 | blocker | 665 | 8078 | 59.1 | 11.20 | mean & SD |
| INVEST | 4.0 | 4 | CCB | 569 | 8098 | 56.0 | 11.80 | mean & SD |
| CAPPP | 6.1 | 3 | blocker | 380 | 5230 | 58.2 | 15.90 | mean & SD |
| CAPPP | 6.1 | 5 | ACE inhibitor | 337 | 5183 | 57.1 | 14.80 | mean & SD |
| LIFE | 4.8 | 3 | blocker | 320 | 3979 | 62.3 | 10.74 | mean & SD |
| LIFE | 4.8 | 6 | ARB | 242 | 4020 | 66.1 | 12.00 | mean & SD |
| VALUE | 4.2 | 4 | CCB | 845 | 5074 | 56.4 | 13.10 | mean & SD |
| VALUE | 4.2 | 6 | ARB | 690 | 5087 | 57.8 | 13.00 | mean & SD |

Before any analysis is done, we need to put our data in a form that BUGSnet can easily handle. This is done by simple running the `data.prep()` function and specifying the name of the treatment, study, and sample size variables:

```
data(diabetes.sim)
rate2.slr <- data.prep(arm.data = diabetes.sim,
                     varname.t = "Treatment",
                     varname.s = "Study")
```

We will use this `data.prep` object throughout the rest of our example.

## Network of Evidence

To generate a network plot, simply input the `data.prep` object into the `net.plot()` function as shown:

```
par(mar=c(1,1,1,1)) #reduce margins for nicer plotting
net.plot(rate2.slr, outcome = "diabetes", node.scale = 3, edge.scale=1.5)
```

Our network has 6 treatments and a high degree of connectedness. If a user wants to highlight a specific node and its direct evidence, this can be done using the `flag` option.

```
par(mar=c(1,1,1,1)) #reduce margins for nicer plotting
net.plot(rate2.slr, outcome = "diabetes", node.scale = 3, edge.scale=1.5, flag = "ARB")
```

This gives us the names of the 5 trials connecting the ARB node to 4 treatments.

### Generate Network Characteristics via `net.tab()`

In addition to a plot of the network, BUGSnet can summarize network, intervention, and comparison statistics that can be used to better understand our network of evidence.

```
network.char <- net.tab(data = rate2.slr,
                        outcome = "diabetes",
                        N = "n",
                        type.outcome = "rate2",
                        time = "followup")
```

#### Network Characteristics

`network.char$network` generates characteristics about the network, such as connectedness, number of treatments in the network, and in the case of a binomial outcome, the number of events in the network.

| Characteristic | Value |
| --- | --- |
| Number of Interventions | 6 |
| Number of Studies | 22 |
| Total Number of Patients in Network | 154176 |
| Total Possible Pairwise Comparisons | 15 |
| Total Number of Pairwise Comparisons With Direct Data | 14 |
| Is the network connected? | TRUE |
| Number of Two-arm Studies | 18 |
| Number of Multi-Arms Studies | 4 |
| Total Number of Events in Network | 10962 |
| Number of Studies With No Zero Events | 22 |
| Number of Studies With At Least One Zero Event | 0 |
| Number of Studies with All Zero Events | 0 |
| Mean person follow up time | 4.06 |

#### Intervention Characteristics

`network.char$intervention` generates outcome and sample size data broken down by treatment.

| Treatment | n.studies | n.events | n.patients | person.time.fup | min.event.rate | max.event.rate | events.per.person | overall.event.rate |
| --- | --- | --- | --- | --- | --- | --- | --- | --- |
| ACE inhibitor | 8 | 1618 | 23351 | 106027 | 0.0072632 | 0.0570593 | 0.0692904 | 0.0152603 |
| ARB | 5 | 1189 | 14185 | 57291 | 0.0051020 | 0.0322952 | 0.0838209 | 0.0207537 |
| blocker | 9 | 2705 | 36150 | 173098 | 0.0057888 | 0.0454841 | 0.0748271 | 0.0156270 |
| CCB | 9 | 2791 | 38809 | 163469 | 0.0094210 | 0.0416884 | 0.0719163 | 0.0170736 |
| Diuretic | 8 | 973 | 18699 | 71931 | 0.0060320 | 0.0408163 | 0.0520349 | 0.0135269 |
| Placebo | 9 | 1686 | 22982 | 89692 | 0.0026489 | 0.0616024 | 0.0733618 | 0.0187977 |

#### Comparison Characteristics

`network.char$comparison` generates outcome and sample size data broken down by treatment **comparison**.

| comparison | n.studies | n.patients | n.outcomes | patient\_time | proportion | event.rate |
| --- | --- | --- | --- | --- | --- | --- |
| ACE inhibitor vs. blocker | 3 | 15158 | 1022 | 409266 | 0.0674231 | 0.0024972 |
| ACE inhibitor vs. CCB | 3 | 12597 | 538 | 289731 | 0.0427086 | 0.0018569 |
| ACE inhibitor vs. Diuretic | 2 | 16488 | 759 | 263808 | 0.0460335 | 0.0028771 |
| ACE inhibitor vs. Placebo | 3 | 17893 | 1929 | 429432 | 0.1078075 | 0.0044920 |
| ARB vs. blocker | 1 | 7999 | 562 | 71991 | 0.0702588 | 0.0078065 |
| ARB vs. CCB | 1 | 10161 | 1535 | 81288 | 0.1510678 | 0.0188835 |
| ARB vs. Diuretic | 1 | 392 | 9 | 784 | 0.0229592 | 0.0114796 |
| ARB vs. Placebo | 2 | 9778 | 573 | 127114 | 0.0586009 | 0.0045078 |
| blocker vs. CCB | 5 | 44974 | 3361 | 1933882 | 0.0747321 | 0.0017380 |
| blocker vs. Diuretic | 2 | 8752 | 241 | 166288 | 0.0275366 | 0.0014493 |
| blocker vs. Placebo | 1 | 3315 | 71 | 36465 | 0.0214178 | 0.0019471 |
| CCB vs. Diuretic | 2 | 15739 | 768 | 220346 | 0.0487960 | 0.0034854 |
| CCB vs. Placebo | 1 | 9711 | 331 | 58266 | 0.0340851 | 0.0056808 |
| Diuretic vs. Placebo | 3 | 7343 | 384 | 198261 | 0.0522947 | 0.0019368 |

## Patient Characteristics

**Within** an RCT, patient characteristics between arms should be well balanced. But **between** RCTs, patient characteristics can vary quite a bit. All potential effect modifiers should be assessed prior to running network meta-analysis. BUGSnet allows for a visual display of potential effect modifiers using `data.plot()`. Here we can see that `age` is well balanced between trials and treatments. Note that an error will be produced due to the fact that standard deviation was not reported in 2 studies (hence the missing error bars in the plot).

```
data.plot(data = rate2.slr,
              covariate = "age", 
              half.length = "age_SD",
              by = "treatment",
              avg.hline=TRUE, #add overall average line?
              text.size = 12) 
#> Warning: Removed 4 rows containing missing values (geom_errorbar).
```

If there are any imbalances in effect modifiers between trials, subgroup analysis or meta-regression can be employed. For this example, we will assume that there is no significant imbalance in effect modifiers.

For a guide on how to perform meta-regression in BUGSnet, consult BUGSnet’s documentation and other vignettes.

## Main analysis

### Model Choice

In the case where study followup time is reported along with the number of events we need to employ the “binomial” family along with the complementary log-log (cloglog) link function. We will run both random and fixed effects models and compare their fit statistics using BUGSnet. Note that both of these models assume a constant hazard ratio for the duration of follow up. A mathematical description of the models follows.

Let \(i=1,...,M\) be labels for the \(M\) studies in the data and let \(t\_{ik}\in\{1,…,T\}\) denote the treatment used in arm \(k\) of study \(i\). The set \(\{1,…,T\}\) represents the set of treatments that were assessed across the \(M\) studies, where treatment 1 is chosen to represent the reference treatment. Let \(R\_{ik}\) be the measured aggregate response in arm \(k\) of study \(i\). Let \(\mathbf R = (R\_{11}, ..., R\_{(1a\_1)}, ..., R\_{M1}, ..., R\_{(Ma\_M)})\top\) be the vector of responses across all study arms, where \(a\_1,...,a\_M\) represent the number of arms in studies \(1,...,M\). The data is modeled using the **binomial family**: \(R\_{ik} \sim Binomial(n\_{ik},\phi\_{ik})\), where \(\phi\_{ik}\) is the probability of experiencing the event in arm \(k\) of study \(i\) and \(n\_{ik}\) is the sample size in arm \(k\) of study \(i\). The latent parameters \(\phi\_{ik}\) are transformed using a **cloglog link** function and modeled with the following linear model:

\[cloglog(p\_{ik})=log(f\_i) +\mu\_i +\delta\_{ik},\]

where \(f\_i\) is the follow-up time in study \(i\), \(\mu\_i\) represents the fixed effect of the treatment from arm 1 in study i (a control treatment) and \(\delta\_{ik}\) represents the (fixed or random) effect of the treatment from arm \(k\) of study \(i\) relative to the treatment in arm 1. Also, \(\delta\_{i1}=0\) for \(i=1,...,M\).

In the random effects model, the \(\boldsymbol \delta\_i’s=(\delta\_{i2},...,\delta\_{ia\_i} )^\top\) are conditionally independent with distributions

\[[\boldsymbol\delta\_i│\boldsymbol d\_i,\Sigma] \sim MVNormal(\boldsymbol d\_i,\Sigma),\]

where \(\boldsymbol d\_i=(d\_{(t\_{i1},t\_{i2})},...,d\_{(t\_{i1},t\_{ia\_i)}})^\top\) and \(d\_{(t\_{i1},t\_{ik})}=d\_{(1,t\_{ik})}-d\_{(1,t\_{i1})}\) is the difference in the treatment effect of treatments \(t\_{i1}\) and \(t\_{ik}\) on the cloglog scale and \(d\_{(1,1)}=0\). For \(\Sigma\), a compound symmetry structure is specified following (16), with variances \(\sigma^2\) and covariances \(0.5\sigma^2\), where \(\sigma^2\) represents the between-trial variability in treatment effects (heterogeneity).

In the fixed effect model, the \(\delta\_{ik}\)’s are treated as fixed (from a frequentist perspective) and are defined as \(\delta\_{ik}=d\_{(t\_{i1},t\_{ik})}=d\_{(1,t\_{ik})}-d\_{(1,t\_{i1})}\) with \(d\_{(1,1)}=0\). Independent priors are specified on \(d\_{(1,2)},...,d\_{(1,T)}\) and \(\mu\_1,...,\mu\_M\). Note that in both fixed and random-effects models, the quantities of interest are \(d\_{(1,2)},...,d\_{(1,T)}\) which allow us to conclude on all treatment contrasts thought the transitivity relation \(d\_{(t\_{i1},t\_{ik})}=d\_{(1,t\_{ik})}-d\_{(1,t\_{i1})}\).

Turning back to the analysis, the first step is to use `nma.model()` to build a model using BUGS code based on the user’s specifications. The `reference` parameter indicates the name of the treatment that will be seen as the ‘referent’ comparator, this is often a placebo of some sort. In our case, we have set the referent treatment to “Diuretic” to be consistent with the TSD and literature.

```
random_effects_model <- nma.model(data=rate2.slr,
                                 outcome="diabetes",
                                 N="n",
                                 reference="Diuretic",
                                 family="binomial",
                                 link="cloglog",
                                 time = "followup",
                                 effects= "random")

fixed_effects_model <- nma.model(data=rate2.slr,
                                 outcome="diabetes",
                                 N="n",
                                 reference="Diuretic",
                                 family="binomial",
                                 link="cloglog",
                                 time = "followup",
                                 effects="fixed")
```

If you want to review the BUGS code that was created, you can use the commands `random_effects_model$model` and `fixed_effects_model$model` (see appendix for an example). Priors are defined on \(d\_{(1,2)},...,d\_{(1,T)}\) and \(\mu\_1,...,\mu\_M\) in both fixed and random effect models, and an additional prior is defined on \(\sigma\) in the random effect model. By default, BUGSnet implements the vague priors proposed in van Valkenhoef, G et al. (2012). You may specify your own priors instead by specifying the options `prior.mu`, `prior.d`and `prior.sigma` in the `nma.model()` function.

Once the NMA models have been specified, the next step is to run the models using `nma.run()` which will analyse the data with the BUGS model through the JAGS software (Plummer 2017). Since we are working in a Bayesian framework, we need to specify the number of adaptations, burn-ins, and iterations. A description of Bayesian MCMC is omitted here, we direct the reader to any introductory text on Bayesian Modelling (Lunn et al. 2012).

```
set.seed(20190828)
fixed_effects_results <- nma.run(fixed_effects_model,
                           n.adapt=1000,
                           n.burnin=1000,
                           n.iter=10000)

random_effects_results <- nma.run(random_effects_model,
                           n.adapt=1000,
                           n.burnin=1000,
                           n.iter=10000)
```

Compare fixed vs random effects models by comparing leverage plots and the DIC. A plot of the \(leverage\_{ik}\) vs \(w\_{ik}\) (Bayesian deviance residual) for each of the \(k\) arms in all \(i\) trials can help highlight potential outliers when fitting our model. If a data point lies outside the purple arc (\(x^2 + y =3\)), then this data point can be said to be contributing to the model’s poor fit.

```
par(mfrow = c(1,2))
fe_model_fit <- nma.fit(fixed_effects_results, main = "Fixed Effects Model" )
re_model_fit <- nma.fit(random_effects_results, main= "Random Effects Model")
```

The random effects model is obviously the better choice here. The DIC is considerably lower in the random effects model. The fixed effects model shows that 8 points are largely contributing to the model’s poor fit. The random effects model appears to have only 1 outlier, which should be investigated. We can see which arm/study is reponsible for this datapoint by using the command `re_model_fit$w`. From this we can see that Placebo arm from the “MRC-E” study is contributing to the model’s poor fit. This may call for a reconsideration of including this study in our evidence base, or to investigate potential effect modifiers. But for the sake of this example, we will assume that this study arm should remain in the evidence base and that there are no imbalanced effect modifiers.

### Check inconsistency

Next, we will assess consistency in the network by fitting a random effects inconsistency model and comparing it to our random effects consistency model (Lu and Ades 2006). If our inconsistency model shows a better fit than the consistency model, then it is likely that there is inconsistency in the network.

```
re_inconsistency_model <- nma.model(data=rate2.slr,
                                   outcome="diabetes",
                                   N="n",
                                   reference="Diuretic",
                                   family="binomial",
                                   link="cloglog",
                                   time = "followup",
                                   type = "inconsistency", #specifies inconsistency model
                                   effects="random")

re_inconsistency_results <- nma.run(re_inconsistency_model,
                                         n.adapt=1000,
                                         n.burnin=1000,
                                         n.iter=10000)
```

Again, rainbow plots and DIC calculations can highlight outliers and can compare model fit between the two models.

```
par(mfrow = c(1,2))
re_model_fit <- nma.fit(random_effects_results, main = "Consistency Model" )
inconsist_model_fit <- nma.fit(re_inconsistency_results, main= "Inconsistency Model")
```

When assessing the fit of both models, the consistency model has a smaller DIC, but the inconsistency model seems to have a neater leverage plot. You’ll notice that there are a couple of other metrics that are outputted with the leverage plots, namely, the leverage (\(pD\)) and the posterior mean of the residual deviance (\(\bar{D}\_{res}\)). The leverage can be thought of as the “effective number of parameters”, while the residual deviance can be thought of as the “model fit” (the lower the better). We can see that the inconsistency model produces slightly a smaller \(\bar{D}\_{res}\), but at the expense of increased model complexity (larger \(pD\)). In general, the consistency model seems to be favourable here, due to it’s adequate fit and parsimony.

Furthermore, a plot of the posterior mean deviance of the individual data points in the inconsistency model against their posterior mean deviance in the consistency model can highlight descrepancies between the two models.

```
nma.compare(re_model_fit, inconsist_model_fit)
```

With the exception of 1 or 2 points, the data lies on or near the \(y=x\) line, indicating a general agreement between the two models. This suggests that we proceed with the more parsimonious (consistency) model.

## Results

### Treatment Rankings

Simulaneous comparison of every treatment based on the results of the NMA analysis can be achieved by comparing the posterior probabilities of being the best, second best,…, worst treatment. In BUGSnet, simply input the results of your model into the `nma.rank()` function, and specify `largerbetter=TRUE` if a larger outcome is associated with better treatments, and `FALSE` otherwise.

The ranking probabilities can be displayed in the form of a rankogram.

```
sucra.out <- nma.rank(random_effects_results, largerbetter=FALSE, sucra.palette= "Set1")
sucra.out$rankogram
```

From this rankogram, we can see that there is a \(\sim\) 77% probability that ARB’s are the best treatments, and a \(\sim\) 80% probability that diuretics are the worst treatment. If you want the exact ranking percentages, these results are also available in tabular form via `sucra.out$ranktable`.

| rank | ACE inhibitor | ARB | blocker | CCB | Diuretic | Placebo |
| --- | --- | --- | --- | --- | --- | --- |
| 1 | 22.92 | 76.41 | 0.00 | 0.08 | 0.00 | 0.59 |
| 2 | 69.55 | 20.80 | 0.01 | 1.99 | 0.00 | 7.64 |
| 3 | 6.83 | 2.45 | 0.08 | 26.53 | 0.01 | 64.10 |
| 4 | 0.69 | 0.33 | 1.38 | 70.21 | 0.34 | 27.04 |
| 5 | 0.01 | 0.00 | 79.86 | 1.13 | 18.39 | 0.61 |
| 6 | 0.00 | 0.00 | 18.67 | 0.06 | 81.26 | 0.01 |

A surface under the cumulative ranking curve (SUCRA) plot can also be displayed via `sucra.out$sucraplot`.

If you want the exact percentages, these results are also available in tabular form via `sucra.out$sucratable`. The last row contains the SUCRA percentages calculated as the average of the cumulative percentages for ranks 1 to 5.

| rank | ACE inhibitor | ARB | blocker | CCB | Diuretic | Placebo |
| --- | --- | --- | --- | --- | --- | --- |
| 1 | 22.92 | 76.41 | 0 | 0.08 | 0 | 0.59 |
| 2 | 92.47 | 97.21 | 0.01 | 2.07 | 0 | 8.23 |
| 3 | 99.3 | 99.66 | 0.09 | 28.6 | 0.01 | 72.33 |
| 4 | 99.99 | 99.99 | 1.47 | 98.81 | 0.35 | 99.37 |
| 5 | 100 | 99.99 | 81.33 | 99.94 | 18.74 | 99.98 |
| 6 | 100 | 99.99 | 100 | 100 | 100 | 99.99 |
| SUCRA | 82.94 | 94.65 | 16.58 | 45.9 | 3.82 | 56.1 |

Since those plots were produced using ggplot2, one can easily customize the plots using ggplot2 commands, for instance via `sucra.out$sucraplot + theme_gray()`:

### League Table and Heatplot

League tables are another great way to summarize the results of an NMA. League tables contain all information about relative effectiveness for all possible pairs of interventions (Rouse, Chaimani, and Li 2017). BUGSnet includes 95% credible intervals. You can also plot the league table as a heatplot using the following code:

```
league.out <- nma.league(random_effects_results,  
                         central.tdcy="median",
                         order = sucra.out$order,
                         log.scale = FALSE,
                         low.colour = "springgreen4",
                         mid.colour = "white",
                         high.colour = "red",
                         digits = 2)
league.out$heatplot
```

Because we used the option `log.scale = FALSE`, the values reported are on the hazard ratio scale as opposed to the log hazard ratio scale. In this example, a green cell indicates that a treatment performed better than its comparator (estimate smaller than 1), while a red cell indicates that the treatment performed worst than its comparator (estimate greater than 1). The symbols (\*\*) are used to highlight credible intervals that do not contain the neutral value 1, meaning that there is evidence of a statistically significant difference between the treatment and its comparator at the 95% confidence level. The values can also be outputted in a table via the command `league.out$table`.

|  | ARB | ACE inhibitor | Placebo | CCB | blocker | Diuretic |
| --- | --- | --- | --- | --- | --- | --- |
| ARB | ARB | 1.07 (0.88 to 1.32) | 1.20 (0.99 to 1.45) | 1.26 (1.06 to 1.54) | 1.49 (1.24 to 1.82) | 1.60 (1.30 to 2.03) |
| ACE inhibitor | 0.93 (0.75 to 1.14) | ACE inhibitor | 1.12 (0.96 to 1.29) | 1.17 (1.01 to 1.39) | 1.38 (1.19 to 1.63) | 1.49 (1.27 to 1.78) |
| Placebo | 0.83 (0.69 to 1.01) | 0.89 (0.77 to 1.04) | Placebo | 1.05 (0.90 to 1.26) | 1.24 (1.05 to 1.48) | 1.33 (1.13 to 1.61) |
| CCB | 0.80 (0.65 to 0.95) | 0.85 (0.72 to 0.99) | 0.96 (0.79 to 1.11) | CCB | 1.18 (1.03 to 1.34) | 1.27 (1.08 to 1.51) |
| blocker | 0.67 (0.55 to 0.80) | 0.72 (0.61 to 0.84) | 0.81 (0.67 to 0.95) | 0.85 (0.74 to 0.97) | blocker | 1.08 (0.91 to 1.29) |
| Diuretic | 0.62 (0.49 to 0.77) | 0.67 (0.56 to 0.79) | 0.75 (0.62 to 0.88) | 0.79 (0.66 to 0.93) | 0.93 (0.78 to 1.10) | Diuretic |

### Forest Plot

Forest plots are another great way to summarize the results of an NMA with respect to a particular comparator.

```
nma.forest(random_effects_results,
           central.tdcy="median",
           comparator = "Placebo",
           log.scale = FALSE)
```

## Conclusion

A random effects model showed to be adequate in modelling the treatment effects across the evidence network. Both the SUCRA plot and league table suggest that all treatments are effective when compared diuretic, with the exception of Beta Blockers. ARB’s seem to be the most effective treatment, followed by ACE inhibitors and Placebo.

## Appendix

### BUGS code for final model

The following BUGS code is produced by the `nma.model()` function.

```
cat(random_effects_model$bugs)
#> 
#>      
#>       # Random effects model for multi-arm trials
#>     
#>       model{                               # *** PROGRAM STARTS
#>   
#>       for(i in 1:ns){                      # LOOP THROUGH STUDIES
#>         w[i,1] <- 0    # adjustment for multi-arm trials is zero for control arm
#>         delta[i,1] <- 0             # treatment effect is zero for control arm
#>         for (k in 1:na[i]) {             # LOOP THROUGH ARMS
#>           rhat[i,k] <- p[i,k] * n[i,k] # expected value of the numerators
#>     dev[i,k] <- 2 * (r[i,k] * (log(r[i,k])-log(rhat[i,k])) #Deviance contribution
#>     + (n[i,k]-r[i,k]) * (log(n[i,k]-r[i,k]) - log(n[i,k]-rhat[i,k])))
#>           # model for linear predictor
#>           cloglog(p[i,k]) <- log(time[i,k]) + mu[i] + delta[i,k]
#>           r[i,k] ~ dbin(p[i,k],n[i,k]) # binomial likelihood
#>         }
#>         resdev[i] <- sum(dev[i,1:na[i]]) #JS
#>         for (k in 2:na[i]) {             # LOOP THROUGH ARMS
#>           # trial-specific LOR distributions
#>           delta[i,k] ~ dnorm(md[i,k],taud[i,k])
#>           # mean of LOR distributions, with multi-arm trial correction
#>           md[i,k] <-  d[t[i,k]] - d[t[i,1]] + sw[i,k]
#>           # precision of LOR distributions (with multi-arm trial correction)
#>           taud[i,k] <- pow(sigma2,-1) *2*(k-1)/k
#>           # adjustment, multi-arm RCTs
#>           w[i,k] <- (delta[i,k] - d[t[i,k]] + d[t[i,1]])
#>           # cumulative adjustment for multi-arm trials
#>           sw[i,k] <- sum(w[i,1:(k-1)])/(k-1)
#>         }
#>       }   
#>       totresdev <- sum(resdev[])
#>       d[1]<-0       # treatment effect is zero for reference treatment
#>       for(i in 1:ns){
#>                                mu[i] ~ dnorm(0,(0.439460189424528*15)^(-2))
#>                                }
#>       for(k in 2:nt){
#>                              d[k] ~ dnorm(0,(0.439460189424528*15)^(-2))
#>     }
#>       sigma ~ dunif(0,0.439460189424528)
#>                                  sigma2 <- sigma^2
#>       tau <- pow(sigma,-2)
#>       
#>     }
#> 
#> # Treatment key
#> # 1: Diuretic
#> # 2: ACE inhibitor
#> # 3: ARB
#> # 4: blocker
#> # 5: CCB
#> # 6: Placebo
```

### Assessment of convergence of final model

After a model is fit to the evidence, check the trace plots of the model using `nma.diag()`. Look for spikes or general irregularities in the posterior distributions of the \(d\)’s. Irregularities in these distributions suggest that the model never converged, and will likely require you to run more have a longer burn-in period, and more iterations. If you wish to know which treatments are causing irregularities, you can use the command `random_effects_model$trt.key` to see which treatment correspond to which integer number on the traceplot. Here, the model shows strong signs of convergence.

```
nma.diag(random_effects_results, plot_prompt = FALSE)
```

```
#> $gelman.rubin
#> Gelman and Rubin's PSRF Convergence Diagnostic:
#> --------------------------------
#>       Point est. Upper C.I.
#> d[2]  1.00       1.00      
#> d[3]  1.00       1.00      
#> d[4]  1.00       1.00      
#> d[5]  1.00       1.00      
#> d[6]  1.00       1.00      
#> sigma 1.00       1.01      
#> --------------------------------
#> Multivariate PSRF: 1.00
#> --------------------------------
#> * PSRF > 1.2
#> 
#> 
#> $geweke
#> Geweke's Convergence Diagnostic Results:
#> --------------------------------
#>       Chain 1 Chain 2 Chain 3
#> d[2]  0.86    -0.38   -1.80  
#> d[3]  0.86    -0.96   -0.71  
#> d[4]  1.13    -0.85   -0.34  
#> d[5]  0.49    -0.87   -0.61  
#> d[6]  0.99    -1.21   -1.71  
#> sigma -1.29   1.16    1.46   
#> --------------------------------
#> Fraction in 1st window = 0.1
#> Fraction in 2nd window = 0.5
#> * Statistically significant at the 95% confidence level
```

# References

Dias, Sofia, Nicky J Welton, Alex J Sutton, and AE Ades. 2011. “NICE Dsu Technical Support Document 2: A Generalised Linear Modelling Framework for Pairwise and Network Meta-Analysis of Randomised Controlled Trials.” National Institute for Health; Clinical Excellence.

Lu, Guobing, and AE Ades. 2006. “Assessing Evidence Inconsistency in Mixed Treatment Comparisons.” *Journal of the American Statistical Association* 101 (474). Taylor & Francis: 447–59.

Lunn, David, Chris Jackson, Nicky Best, David Spiegelhalter, and Andrew Thomas. 2012. *The Bugs Book: A Practical Introduction to Bayesian Analysis*. Chapman; Hall/CRC.

Plummer, Martyn. 2017. *JAGS Version 4.3.0 User Manual*. http://people.stat.sc.edu/hansont/stat740/jags\_user\_manual.pdf.

Rouse, Benjamin, Anna Chaimani, and Tianjing Li. 2017. “Network Meta-Analysis: An Introduction for Clinicians.” *Internal and Emergency Medicine* 12 (1). Springer: 103–11.

van Valkenhoef, G, Lu, G, de Brock, B, Hillege, H, Ades, AE, and Welton, NJ. 2012. “Automating Network Meta‐analysis.” *Research Synthesis Methods* 3 (4): 285–99.
